# Latent classes of eating disorders and addictions by sex: Implication of alexithymia and stressful life events in youths

**DOI:** 10.3389/fpsyg.2023.1088595

**Published:** 2023-02-10

**Authors:** Laura Macía, Janire Momeñe López, Patricia Macía, Marta Herrero, Paula Jauregui, Iciar Iruarrizaga, Ana Estévez

**Affiliations:** ^1^Psychology Department, School of Health Sciences, University of Deusto, Bilbao, Spain; ^2^Department of Basic Psychological Processes and their Development, University of the Basque Country/Euskal Herriko Unibertsitatea (UPV/EHU), Donostia-San Sebastián, Spain; ^3^Department of Experimental Psychology, Cognitive Processes and Speech Therapy, Faculty of Social Work, Complutense University of Madrid, Madrid, Spain

**Keywords:** eating disorder, addiction, gambling, alexithymia, stressful life events, sex differences

## Abstract

**Introduction:**

Eating disorders (EDs) and behavioural addictions show common psychological vulnerability factors such as alexithymia and stressful life events (SLE). This study aims, firstly, to explore the prevalence and latent profiles of participants based on their risk of suffering EDs, gambling disorder (GD), alcohol and/or drug abuse, and compulsive buying (CB) by sex. Secondly, it aimed to test whether alexithymia and having experienced SLE are associated with group membership.

**Methods:**

The sample was predominantly drawn from university students and social networks. It was composed of 352 young adults between 18 and 35 years old, of whom 77.8% were women and 22.2% men.

**Results:**

The results showed that the most prevalent disorders of the sample were alcohol, EDs, CB, drugs and GD, respectively. Moreover, latent class analyses were conducted based on the risk of suffering EDs or addictions by sex. Three main profiles were found: ‘Men with addictions’, ‘Healthy women’ and ‘Women with EDs’. Finally, differences in SLE and alexithymia levels were tested by latent classes. “Men with addictions” and “Women with EDs” had higher scores on alexithymia and SLE than the group of “Healthy women”. However, the group of “Women with EDs” (class 3) reported significantly higher levels of SLE and alexithymia than the other two groups.

**Discussion and conclusion:**

In conclusion, we discuss the possibility that some vulnerability factors operate generally and transdiagnostically in EDs and addictive disorders. The identification of clinical phenotypes could complement and deepen prediction, prevention and treatment research in clinical settings. The need to take sex and gender differences into account is reinforced.

## Introduction

The complex heterogeneity of comorbid mental health disorders, such as alcohol use disorders, drug addiction, gambling disorder (GD), compulsive buying (CB), or eating disorders (EDs) makes it necessary to explore the main underlying transdiagnostic risk factors that could lead to these mental health conditions (Crews and Boettiger, 2009; Romer, 2010; Lavender and Mitchell, 2015; Rømer Thomsen et al., 2018; Brunelle and Grossman, 2022). Research should be directed to clarify common causes and similar development patterns of addictive behaviours and EDs, in order to identify specific characteristics of subgroups of patients and, in turn, clinical implications for prevention and intervention.

Gambling disorder was the first behavioural addiction recognised within the diagnostic category of ‘Substance-Related and Addictive Disorders’ in the fifth edition of the *Diagnostic and Statistical Manual of Mental Disorders* [DSM-5; American Psychiatric Association (APA), 2013]. Currently, GD is the only behavioural addiction recognised in DSM-5, despite widespread interest among researchers and health professionals in behaviours, such as CB, ‘food addiction’, ‘sexual addiction’, ‘exercise addiction’ or ‘internet addiction’, among others. However, further empirical evidence is still needed to determine the transferability of DSM-5 criteria to other addictive behaviours (Potenza, 2014; Robbins and Clark, 2015).

A recent study has reported that the prevalence of GD in Spain is 0.72%, which is now higher than that indicated in the DSM-5 [0.2–0.3%; American Psychiatric Association (APA), 2013; Chóliz et al., 2021]. A systematic review of worldwide GD prevalence estimates it between 0.12 and 5.8% (Calado and Griffiths, 2016), while the estimated prevalence of young people meeting the criteria for GD varies from 0.2 to 12.3% (Calado et al., 2017). Existing evidence also indicates that online gambling is the leading cause of GD in the youth population (Chóliz, 2016). Men show higher prevalence of GD than women (Wong et al., 2013). However, some studies have identified greater severity of GD in women than in men (Grant et al., 2012; Merkouris et al., 2016; Chóliz et al., 2021).

Regarding CB, a meta-analysis by Maraz et al. (2016) estimated a pooled prevalence of 4.9%, showing a higher trend among younger ages and females (Granero et al., 2016; Estévez et al., 2020). However, other studies of the area have shown a similar prevalence among men and women (Koran et al., 2006), suggesting that the sex difference lies in the types of goods purchased (Hayhoe et al., 2000). However, there are still not enough studies incorporating a gender perspective to adequately interpret the results of either GD or CB.

The national Spanish survey on the prevalence of alcohol and other drugs showed that alcohol is currently the most widely consumed psychoactive substance and the one with the earliest onset of use, along with tobacco. Regarding illicit drug use, cannabis is the most consumed substance, followed by cocaine. Meanwhile, the prevalence of prescribed and non-prescribed hypnosedatives continued to increase over the last decade, and it is the only substance that is more prevalent among women than among men (Delegation of the Spanish Government for the National Plan on Drugs, 2020).

Behavioural addictions often co-exist with other psychiatric disorders such as EDs (Starcevic and Khazaal, 2017). EDs are characterised by a persistent disturbance of eating behaviour. The three main types of EDs are anorexia nervosa (AN), bulimia nervosa (BN) and binge eating disorder (BED; Hay, 2020). Recent studies indicated that weighted means of lifetime EDs were 8.4% for women and 2.2% for men, tending to emerge between early and late adolescence (Galmiche et al., 2019; Momeñe et al., 2022a). However, EDs incidence continues to increase, becoming a genuine concern for mental health services, especially among young girls.

Several studies have reported that CB and EDs are highly comorbid. In this line, a study by Munguía et al. (2021b) reported that the co-occurrence of CB and EDs leads to a more severe clinical profile and a worse treatment outcome when compared to healthy controls or patients without this comorbidity (de Mattos et al., 2018). In contrast, the prevalence of GD and EDs appears to be lower (Jiménez-Murcia et al., 2013). One important epidemiologic consideration for these results is the impact of sex. That is, the expectation of alleviating a negative affect through behaviour (‘negative urgency’) has been found to be strongly related to dysfunctional eating behaviours in women, whereas dysfunctional gambling activities have been associated with men (Fischer and Smith, 2008). However, the very fact that EDs are more common in women and GD is more common in men has implied a limited awareness of the comorbidity of these conditions. In fact, dual diagnosis studies of patients suffering from GD and EDs suggest clinically relevant differences (Jiménez-Murcia et al., 2015). More specifically, the clinical picture of GD + EDs appears to be more severe, with a higher tendency towards alcohol and drug abuse, depressive disorders, bipolar disorders, anxiety disorders, personality disorders, impulsivity, suicidality and more gambling-related cognitive distortions (Kim et al., 2018; Lemón et al., 2021).

The increased comorbid presence of EDs and behavioural addictions shows common psychological vulnerability factors that could require specific clinical attention (Fernández-Aranda et al., 2019). Moreover, it has been observed that even if there is no direct relationship between EDs and addictive pathologies, some common risk traits are likely (von Ranson et al., 2013; Munguía et al., 2021a).

There is an extensive database of study results in the literature that associates alexithymia and trauma with a tendency towards addictions and EDs (Zdankiewicz-Ścigała and Ścigała, 2020; Momeñe et al., 2022b). It has been conceptualised that early trauma disturbs the development of cognitive and affective processing, integration of thinking and feeling, and the capacity to understand and express emotional states (Craparo et al., 2014). Several researchers have posited that stressful life events play a central role in the development of alexithymia (Morie et al., 2020; Zdankiewicz-Ścigała and Ścigała, 2020).

Alexithymia is a multifaceted personality trait first described by Sifneos (1973) that involves difficulties in the identification and description of one’s own and other’s emotions, trouble distinguishing between feelings and bodily sensations of emotional arousal, and an externally orientated thinking style. For example, excessive food intake, excessive alcohol or drug use, CB or GD would be associated with a lack of the capacity to think and express affects. Consequently, it could lead to a reliance on immediate stimuli and the development of non-verbal compensatory strategies to escape, dampen or manage intolerable emotions (Van Strien and Ouwens, 2007; Marchetti et al., 2019; Herman et al., 2020).

### Objectives

In recent years, this line of research examining the relationship between alexithymia and stressful life events regarding addictive behaviours and EDs has gained relevance due to their increased prevalence, clinical and prognostic implications, particularly among young people. Previous studies have not analysed differences in impulse control disorders based on the risk of suffering from them. Furthermore, to the best of our knowledge, the scientific literature does not provide information about how stressful life events and alexithymia influence the development of different addictive and ED profiles, at least in an interrelated and non-segmented way. For this reason, the main objective of this study is to explore the prevalence and latent profiles of participants based on their risk of suffering EDs, GD, alcohol, and/or drug abuse and CB behaviour by sex, in a sample of young adults between the ages of 18 and 35. It also aimed to analyse whether alexithymia and having suffered stressful life events were associated with latent class membership based on risk of addictions and/or EDs and according to sex.

### Hypothesis

- Girls would show higher scores in EDs and CB, while boys would show higher scores in addictive behaviours, particularly in GD and drugs.
- It is expected to find a final model of two or three latent classes.
- Participants who score high in addictions and/or EDs would show higher scores in alexithymia and would have experienced more stressful life events.
- Alexithymia and stressful life events will be associated with latent class profiles with the highest risk of EDs and/or addictions.

## Method

### Participants

Three hundred and 52 young adults participated in the study. The inclusion criterion was to be between 18 and 35 years old (*M* = 22.93, SD = 3.30). In contrast, the only exclusion criterion to participate was being under 18 years old. Most of the sample were women (77.8%; men: 22.2%), and most had university studies (72.7%). Regarding occupational status, 51.4% were students, 25% were students and workers and 20% were only workers.

The sample was recruited through three main ways. On the one hand, students from the Complutense University of Madrid were asked to participate. Students were from degrees related to health and social sciences. On the other hand, data were collected using non-probabilistic sampling through the snowball method. That is, the researchers of the study disseminated the objective of the research and the link to the questionnaire in their close social and professional environment, making the latter do the same with their environment, successively. In third and last place, the questionnaire was also diffused on social networks (e.g., WhatsApp, Instagram, email, Facebook, or LinkedIn), as well as university bulletin boards, journals and websites with divulgation purposes and through the official college of psychologists. No compensation was offered.

### Instruments

#### Impulse control disorders and addiction

The MULTICAGE CAD 4 (Pedrero et al., 2007) is formed by 32 items that assess eight impulse control disorders and addictions (with four items in each factor): Drug and Alcohol Use Disorder (Items 1–4), Gambling Disorder (Items 5–8), Substance addictions (Items 9–12), Eating Disorders (Items 13–16), Internet Addiction (Items 17–20), Gaming Addiction (Items 21–24), Compulsive Spending (Items 25–28) and Sex Addiction (Items 29–32). In this study, we used four of those eight factors (i.e., Alcohol Abuse, Drugs, Compulsive Spending and Eating Disorders). Each subscale is evaluated by reproducing the CAGE scheme (Hayfield et al., 1974): self-perception of the problem, perception by cohabitants, associated feelings of guilt, and signs of abstinence or inability to control the behaviour. The questionnaire is rated on a dichotomous scale ‘*Yes/No*’, in which none or one affirmative answer indicate ‘*no problem*’, two affirmative answers indicate the ‘*possible existence of the problem*’, three affirmative answers indicate ‘*very probable existence of the problem*’ and four affirmative answers indicate ‘*sure existence of the problem*’. Accordingly, the risk of each addiction was determined on the basis of three risk groups. Scores between 0 and 1 were categorised as ‘*non-risk group*’, scores of two points were categorised as ‘*probable risk group*’, and scores between 3 and 4 were categorised as ‘*quite probable risk*’ (see Table 1). The cut-off point was set by the authors at two affirmative responses to indicate problem behaviour and/or presence of addiction. Cronbach’s alpha for the total original scale was 0.86. For the present study, the internal consistency of the four subscales used were: eating disorders *α* = 0.60, alcohol addiction *α* = 0.60, drug addiction *α* = 0.63 and compulsive buying *α* = 0.67.

**Table 1:** Percentages and number of participants per category of eating disorder and addictions.

	Eating disorder			Alcohol			Drugs			Compulsive buying			Gambling	
	No risk	Probable risk	Quite probable risk	No risk	Probable risk	Quite probable risk	No risk	Probable risk	Quite probable risk	No risk	Probable risk	Quite probable risk	No risk	Risk
*n*	261	49	19	232	70	35	298	24	14	284	39	15	320	32
%	79.3	14.9	5.8	68.8	20.8	10.4	88.7	7.1	4.4	84.1	11.5	4.4	90.9	9.1

#### Gambling disorder

The *South Oaks Gambling Screen* (SOGS) questionnaire was used (Lesieur and Blume, 1987) in its Spanish version (Echeburúa et al., 1994). It is a 20-item screening questionnaire based on the DSM-III (American Psychiatric Association, 1980) and DSM-III-R diagnostic criteria for pathological gambling (American Psychiatric Association, 1987). The scale contains items related to gambling patterns, sources of money to gamble or pay debts and emotions involved. The response format consists of a ‘*Yes/No*’ dichotomous scale. Scores above four points suggest the possible presence of GD. The instrument presents good psychometric properties, showing an appropriate internal consistency for the original scale (*α* = 0.94), and the convergent validity with DSM-IV criteria was 0.94. In the present study, Cronbach’s *alpha* for the SOGS was 0.60.

#### Alexithymia

The *Toronto Alexithymia Scale-20* (TAS-20; Taylor et al., 1985) was used to measure alexithymia in its Spanish version (Martínez-Sánchez, 1996). The scale contains 20 items that measure alexithymia through three main factors: (1) Difficulty Identifying Feelings, which refers to problems identifying emotions, with some confusion with physical symptoms, (2) Difficulty Describing Feelings, referring to the inability to communicate one’s feelings and use emotional vocabulary, and (3) Externally-Oriented Thinking, in which individuals tend to neglect their inner emotional states. The scale is rated on a six-point Likert scale ranging from zero (*strongly disagree*) *to* five (s*trongly agree*). The internal consistency is adequate for the overall scale (*α* = 0.83 for the original scale and 0.81 for the Spanish adaptation). In the current study, Cronbach’s alpha was 0.85.

#### Stressful life events

The *Centrality of Event Scale* (CES; Berntsen and Rubin, 2006) was used to assess stressful life events. It was adapted to Spanish by Fernández-Alcántara et al. (2015). The scale measures aspects related to how individuals process traumatic events. Before answering the scale, participants were asked to write down the most traumatic or stressful event they had ever experienced, to base the response on that event when completing the scale. The CES contains 20 items rated on a five-point Likert scale ranging from one (*strongly disagree*) to five (*strongly agree*). The internal consistency is satisfactory both for the original scale and the Spanish adaptation (*α* = 0.94). In this study, Cronbach’s alpha was 0.93.

### Procedure

This study used a cross-sectional research design. All participants completed the survey online (through a link to the questionnaire) or *via* a QR code that gave access to the same questionnaire. To access the questionnaire, participants had to be over 18 years old, have read the study information, and provide informed consent. Completing the questionnaire lasted around 30 min.

Before completing the questionnaire, all participants received general information about the main objectives of the research study. It was made clear that there were no right or wrong answers, and they could contact the main researcher by mail if they needed further information about the study. Confidentiality, anonymity and voluntary participation were ensured for all participants, and they did not receive any compensation for participating. The Institutional Review Board approved the study. This study was performed in line with the principles of the Declaration of Helsinki.

### Statistical analysis

Before conducting the main analysis, patterns of missing data were explored through the MCAR Little test. Then, descriptive analyses were conducted to explore the prevalence of eating disorders and different addictions (in percentages). Secondly, main analyses focused on the latent groups of participants based on the risk of suffering EDs or addictions. Specifically, latent class analyses were conducted (LCA) with Mplus 7.11 (Muthén and Muthén, 1998–2013). In doing so, the variables of sex, risk of EDs, CB, GD, alcohol, and drugs addiction were included in the examination of the latent classes.

In this way, latent class models were compared (with odds ratios tests), and a final model was estimated. The adequacy of the models was based on several model fit indicators and model test comparisons. Specifically, we used Akaike’s Information Criterion (AIC), the Bayesian Information Criterion (BIC), the mean-adjusted Bayesian Information Criterion (aBIC), entropy, the Lo–Mendell–Rubin adjusted likelihood ratio test (LMRa) and the bootstrap likelihood ratio test (BLRT). Lower values of AIC, BIC and aBIc indicate better fit (Hu and Bentler, 1999). Entropy indicates the level of precision of profile classification in values from 0 to 1, so values closer to one are indicators of higher accuracy (Celeux and Soromenho, 1996). The LMRa and BLRT are loglikelihood tests that compare a model with the model with one less class, so significant results indicated a significant increment of model fit for the model with one more class (Nylund et al., 2007).

Finally, ANCOVAs were computed to test the differences in alexithymia and stressful life events between the class membership controlling by age. Pair comparisons between classes were examined with Bonferroni correction.

## Results

Before conducting the analyses, the MCAR Little test was applied to test the patterns of missing data. The test was not significant [*χ*^2^_(987)_ = 1054.04, *p* = 0.068], so it was assumed that missing data were completely at random, and analyses were developed by pairwise deletion.

The initial analyses were conducted to explore the prevalence of EDs and addictions. As displayed in Table 1, approximately two out of 10 participants were at risk of EDs. The most prevalent risk of addiction was alcohol consumption, with almost one-third of the participants at some risk. Drugs and CB were less prevalent, as a smaller amount of 15% of the participants presented some risk. GD was the least prevalent and was present in one out of every 100 participants.

The primary analyses were focused on exploring the latent groups of participants based on their risk of EDs and addictions through latent class analysis (LCA). The first step of the LCA procedure was to examine the number of latent classes comparing models with an incremental number of classes (i.e., latent groups), including risk of EDs and additions in these models.

The models with 1–4 latent classes were compared (see Table 2). The results showed that the 2-class model had a better model fit indicator than the 1-class model, and it significantly increased model fit based on the LMRa and BLRT. The 3-class model had better AIC, BIC, aBIC, and entropy than the 2-class model. Also, the LMRa and BLRT were significant, showing that the 3-class model had a significantly better fit than the 2-class model. Finally, the 4-class model was compared to the 3-class model. In this case, the inclusion of one more class in the model worsened all model fit indicators, and the LMRa and BLRT showed no significant increment in the model fit of the 4-class model compared to the 3-class model. Therefore, the 3-class model was retained as the final model.

**Table 2:** Comparison of latent class models.

Model	AIC	BIC	aBIC	Entropy	LMRa	BLRT
1-Class model	2221.89	2260.53	2228.80	–	–	–
2-Class model	2145.67	2260.80	2160.18	0.77	96.72^***^	98.22^***^
3-Class model	2128.34	2251.97	2150.46	0.81	38.73^***^	39.33^***^
4-Class model	2134.33	2300.46	2164.05	0.82	15.76	16.01

AIC, Akaike’s Information Criterion; BIC, Bayesian Information Criterion; aBIC, sample-size-adjusted Bayesian Information Criterion; LMRa, Lo–Mendell–Rubin adjusted likelihood ratio test of K—1 vs. K Profiles; and BLRT, bootstrap likelihood ratio test of K—1 vs. K Class.

*** *p* < 0.001.

The second step of the LCA procedure was to observe the composition of each of the three classes of the final model attending to the risk prevalence of EDs and addictions in each class. The composition of each class is presented in Table 3. All participants in Class 3 had some risk of EDs, were all women, and had no risk of addiction to drugs. Class 3 had a higher risk of GD as well as of CB than Class 2. Compared to Class 1, Class 3 showed lower risk of all addiction risks (i.e., alcohol, drugs and GD) but similar levels of CB.

**Table 3:** Final model estimates in percentage scale and comparisons between classes.

Variable	Class 1 (*n* = 45)	Class 2 (*n* = 249)	Class 3 (*n* = 58)	Comparisons					
				Class 1 vs. 2		Class 1 vs. 3		Class 2 vs. 3	
				OR [95% CI]	*η*	OR [95% CI]	*η*	OR [95% CI]	*η*
Sex									
Male	62.2	17.6	0.0	7.67^**^ [3.87, 15.22]	0.37	95.53^**^ [12.10, 754.49]	0.67	12.45^*^ [1.68, 92.30]	0.18
Female	37.8	82.4	100.0						
Eating disorder									
No risk	74.3	97.2	0.0	0.08^**^ [0.03, 0.22]	0.35	147.23^**^ [18.42, 1,176,58]	0.74	1754.50^**^ [215.17, 14360.23]	0.91
Probable risk	25.7	2.8	60.1	12.25^**^ [4.51, 33.27]	0.34	0.24^**^ [0.11, 0.56]	0.33	0.02^**^ [0.01, 0.05]	0.65
Quite probable risk	0.0	0.0	39.9						
Alcohol									
No risk	19.5	78.2	77.9	0.07^**^ [0.03, 0.15]	0.45	0.07^**^ [0.03, 0.17]	0.59	0.94 [0.47, 1.90]	0.01
Probable risk	52.0	15.3	12.7	5.81^**^ [2.94, 11.45]	0.32	7.62^**^ [2.85, 20.35]	0.43	1.31 [0.55, 3.11]	0.04
Quite probable risk	28.5	6.5	9.4						
Drugs									
No risk	53.0	94.8	100.0	0.07^**^ [0.03, 0.15]	0.45	0.04^**^ [0.01, 0.18]	0.52	0.58 [0.13, 2.63]	0.04
Probable risk	21.8	5.2	0	5.21^**^ [2.12, 12.78]	0.22	16.86^**^ [2.07, 137.35]	0.33	3.24 [0.42, 25.24]	0.07
Quite probable risk	25.2	0.0	0						
Compulsive buying									
No risk	59.2	92.0	72.9	0.13^**^ [0.06, 0.28]	0.34	0.57 [0.25, 1.31]	0.13	4.36^**^ [2.09, 9.10]	0.24
Probable risk	26.5	7.3	15.6	4.67^**^ [2.06, 10.56]	0.23	1.98 [0.75, 5.23]	0.14	0.42*^t^* [0.18, 1.00]	0.12
Quite probable risk	14.3	0.7	11.5						
Addiction to gambling									
No risk	57.3	100.0	86.15	181.96^**^ [23.40, 1414.95]	0.60	4.57^**^ [1.76, 11.84]	0.32	0.03^**^ [<0.01, 0.21]	0.31
Risk	42.7	0.0	13.5						

OR, Odd ratio. For the estimation of OR with zero cells, a fixed value (0.5) was added.

^*^p < 0.05, ^**^p < 0.01.

Class 1 was the only group with a higher presence of men, as men were more than 1.5 times more likely than women to belong to Class 1. Women were significantly more prevalent in Class 2 than in Class 1 and represented eight out of 10 participants. Class 1 had a higher risk of ED, alcohol, drugs, CB and GD than Class 2.

Based on these results, Class 2 was described as a group represented mainly by women without EDs or addictions, so it was named ‘Healthy women’. The great majority of the participants belonged to this group (70.7%). Class 3 was described as a group of women with EDs who were at risk for alcohol, GD and CB, but not for addiction to drugs. Therefore, this group was named ‘Women with EDs’. This group comprised 16.5% of the sample. Class 1 was mainly represented by men at risk for all the measured additions but without EDs. Consequently, this group was named ‘Men with addictions’. This group comprised 12.7% of the sample.

The third step of the LCA procedure was to test the differences in the levels of stressful life events and alexithymia by latent classes (i.e., healthy women, women with EDs and men with addictions). The results of these analyses revealed significant differences between classes in both variables (see Table 4). Concretely, regarding alexithymia, the group of Women with EDs (Class 3) showed significantly higher levels of alexithymia than the group of Healthy women (Class 2; *p* = 0.002) or the group of Men with addictions (Class 1; *p* = 0.010). There were no significant differences in alexithymia between the group of Men with addictions (Class 1) and the group of Healthy Women (Class 2; *p* = 0.592).

**Table 4:** Alexithymia and stressful life events by classes.

Variable	Descriptive statistics						ANCOVA comparison between classes^a^	
	Class 1 men with addiction (*n* = 45)		Class 2 healthy women (*n* = 249)		Class 3 women with eating disorders (*n* = 58)			
	*M*	SE	*M*	SE	*M*	SE	*F*	*p*
Alexithymia	53.94	14.61	46.42	14.50	59.00	16.18	9.46	<0.001
Stressful life events	3.66	0.69	3.30	0.77	3.74	0.60	7.06	0.001

a Age was included as control variable.

Regarding stressful life events, Women with EDs (Class 3) reported significantly higher level of stressful life events than the group of Healthy women (Class 2; *p* = 0.031) or the group of Men with addictions (Class 1; *p* = 0.009). There was no significant difference between the group of Men with addictions (Class 1) and Healthy women (Class 2; *p* = 1.00).

## Discussion

The present study first explored the prevalence of EDs and addictive behaviours in youth population. Results show that the most prevalent disorders are alcohol, EDs, CB, drugs and GD, respectively. The empirical literature has shown that EDs and CB are more prevalent in women, whereas GD and drugs are more prevalent in men (Granero et al., 2016; Chóliz et al., 2021; Momeñe et al., 2022a). Regarding alcohol, although the prevalence tends to be higher in men, both sexes show risky drinking patterns during youth (Delegation of the Spanish Government for the National Plan on Drugs, 2020). However, there are studies with contradictory results, showing greater tendency for young women to drink alcohol, especially binge drinking (White, 2020). In this study, the sample was predominantly comprised of women, so the prevalences found are consistent with our hypotheses and previous literature in the area. Our results add to the evidence that sex and gender roles play an important role in the prevalence of specific mental health conditions and associated attitudes (Rodríguez et al., 2019; Bacigalupe et al., 2020). In this line, it has been argued that girls tend to transgress rules when this is somehow socially permitted or justified (e.g., binge drinking in adolescence and youth stages of life) and only with some types of substances or behaviours (the socially accepted or legalised ones, such as alcohol, overeating or buying; Martínez-Redondo and Luján-Acevedo, 2020).

Secondly, latent class analysis was carried out based on the risk of suffering an ED and/or addictive disorders (i.e., alcohol, drugs, GD and CB) by sex. Moreover, this study aimed to test whether alexithymia and having experienced certain stressful or traumatic life events were associated with group membership. Results have shown the existence of three main profiles. These profiles were: the first group, which was called ‘*Men with addictions’* (Class 1), the second group, named ‘*Healthy women’* (Class 2), and a third group, called ‘*Women with EDs’* (Class 3).

The first group, *‘Men with addictions’* (*Class 1*), was composed mostly of males with high-risk behaviours of drug consumption, alcohol, GD and CB, but no risk of EDs. This group reported having experienced stressful life events, as well as high scores on alexithymia (although lower than the group of women with EDs). The second group, *‘Healthy women’ (Class 2)*, consisted of the majority of the study sample, and was overwhelmingly female and with no risk of either addictive behaviours or EDs. Scores in alexithymia and stressful life events were the lowest of the three groups. The third group, *‘Women with EDs’ (Class 3)*, consisted only of females at high risk of EDs, with a tendency to dysfunctional alcohol use patterns, CB and gambling activities, but without drug use. This group exhibited the highest levels of alexithymia, as well as having suffered stressful life experiences. In fact, ‘Women with EDs’ (Class 3) reported significantly higher levels of stressful life events and alexithymia than the group of ‘Healthy women’ (Class 2) and the group of ‘Men with addictions’ (Class 1).

These results align with previous findings indicating the importance of alexithymia and stressful life events in explaining the onset and maintenance of impulsive risk-taking behaviours (i.e., EDs or addictions; White et al., 2018; Marchetti et al., 2019; Herman et al., 2020). A model proposed by Blaszczynski and Nower (2002) theorises that GD arises from early life stressors, suggesting that engaging in maladaptive behaviours is a way of coping with stress and emotional issues (Nower and Blaszczynski, 2004). In this sense, individuals who experience affective overload have been found to be more vulnerable to developing compensatory non-verbal strategies. It has also been argued that when people are faced with traumatic life events, they focus primarily on survival and self-protection, which could have profound effects on their emotion regulation and coping capacity (van der Kolk, 2000). That is, they may be at a higher risk of developing a coping style in which the goal is taking action rather than understanding and expressing those emotional states (Zdankiewicz-Ścigała and Ścigała, 2020).

In this vein, people with alexithymic traits generally attempt to regulate their emotional states through impulsive behaviours rather than cognitively (Shishido et al., 2013). Impaired emotion regulation has been extensively linked to the aetiology of compulsive behaviours among alexithymic individuals (e.g., binge eating, drug and/or alcohol addiction or GD; Orsolini, 2020). Studies have supported the hypothesis that alexithymia may constitute a stable personality trait, strongly influenced and caused by many factors, including stressful life and early traumatic experiences, which may, in turn, lead to increased vulnerability both to EDs and addictive disorders (Lane et al., 2000; Lyvers et al., 2019; Shank et al., 2019; Lenzo et al., 2020). According to Kaur (2019), individuals who experienced more stressful childhood experiences and reported less ability to identify their moods (i.e., alexithymia) were more likely to use an addictive behaviour as a maladaptive coping mechanism. In addition, posttraumatic disorders are significantly more recurrent in young people in treatment for addiction and EDs than those without them, and are associated with a costlier treatment and worse prognosis (Wang et al., 2020; Momeñe et al., 2022a).

Therefore, in view of the above, the results of the present study highlight the importance of examining stressful life events and alexithymia as precipitating and/or maintenance factors for engaging in risky behaviours among young people. Latent class analysis highlights that experiencing stressful life events and alexithymia may play a central role in the aetiopathogenesis, maintenance and relapses of eating and addictive disorders (Brewerton and Brady, 2014). In fact, the two risk groups (i.e., ‘Men with addictions’—Class 1 and ‘Women with EDs’—Class 3) reported higher scores on both factors (stressful life events and alexithymia) compared to the group with no risk of either addictions or EDs (*‘Healthy women’*—Class 2). Moreover, our findings suggest that both factors are of particular relevance in the case of young women with EDs (Class 3). These results are in line with previous studies indicating that women experience more stressful life events during the life cycle, which may also be influencing their cognitive vulnerability (Hankin and Abramson, 2001). However, given that men with addictions also exhibited elevated means for both alexithymia and stressful life events, we suggest conducting studies with a larger sample of men in the future.

In conclusion, this could lead us to consider that vulnerability factors such as alexithymia and stressful life events operate generally and, therefore, the symptomatic expression and development of one of the disorders (i.e., EDs, CB, GD, alcohol or drugs addiction) is likely to be highly influenced by the associated gender roles. This may contribute to explain why, in the group analysis, EDs were found in the all-female group (Class 3), whereas drug use was found in the mostly-male group (Class 1). In the same vein, we note that in the present study, which was comprised of a predominantly female sample, the highest prevalence was shown for EDs, CB or alcohol, disorders that have been found to occur more frequently in women than in men, in both adult and young populations, as well as in clinical and general populations (Davenport et al., 2012; Otero-López and Villardefrancos, 2014; Maraz et al., 2016; Mestre-Bach et al., 2017). By contrast, GD and drugs were the least prevalent in the study sample, which comprised very few men.

## Limitations and future lines

This study presents some limitations. Firstly, the study employed a cross-sectional design, which precludes establishing the direction of the associations and interpreting results in terms of causality. Longitudinal studies are needed in future studies to study in depth the interactions among variables of interest. In this regard, future lines should explore whether or not stressful life events precede addictive behaviours, including if there are different profiles depending on stressful life events occurring before or after problematic behaviours, and also if there is a difference in the interaction of the variables between EDs and addictions. Likewise, identifying which are those specific stressful or traumatic events in the individual’s life could be essential to fully understanding possible predisposing, precipitating, and/or perpetuating risk factors.

Secondly, it is recommended to homogenise the sample, increasing the number of male participants in the study. Furthermore, results in this study are based on sex differences, so it would be appropriate to conduct future studies focused on gender differences. This would allow exploring and better understanding social conditions and determinants, as well as the differential needs of women and men when facing addiction. Nevertheless, we have discussed the main results based on gender analyses, to include socially constructed attributes for women and men to interpret the results obtained.

Thirdly, the MULTICAGE-CAD 4 and the SOGS scales showed low internal reliability in the present study, with Cronbach’s alpha values under 0.70. This may be due to the dichotomous response of the items which may reduce the Cronbach alpha values (Greer et al., 2006; Adamson and Prion, 2013). As well, the mixed administration method, the continent in which the sample was recruited and the non-clinical sample may explain the low values observed also in other studies (Esparza-Reig et al., 2021). Therefore, the results should be interpreted with caution and replicated with other samples and measures.

Fourthly, we note that some of the questionnaires (i.e., MULTICAGE-CAD 4) are screening tests. Therefore, it would be advisable to administer other types of complementary tests to confirm a possible diagnosis. However, screening is useful for detecting possible pathological behaviours in the general population. Moreover, considering the mean age of the participants and that there are participants from university and social networks, the results are not generalizable to older people and other diagnoses or contexts. However, the subclinical sample of this study does allow us to observe vulnerability factors in youths, which may precede the development of more severe addictive behaviours or Eds in adulthood. This is particularly beneficial for preventive purposes.

## Conclusion and clinical implications

Taken together, this study demonstrates the importance of researching the impact of stressful life events and alexithymia in the development and early recognition of EDs and addictive disorders in the young population, especially among women at risk of EDs. Transdiagnostic approaches to mental health that include the identification of common risk factors, such as alexithymia or having experienced stressful life events, could support the identification of clinical phenotypes to complement and deepen prediction, prevention and/or treatment research in clinical settings.

From a biopsychosocial model, we discuss the possibility that some vulnerability factors operate generally and transdiagnostically in EDs and addictive disorders. Accordingly, the development of one of the disorders could be shaped by the individual’s specific learning history and gender roles, suggesting a more general framework for integrating dispositional and learned risk factors. However, more research is needed to clarify these issues. In particular, future areas of investigation could explore how gender roles influence the development of mental disorders, as well as the influence of pathology-specific risk factors.

In summary, therapeutic techniques that promote mentalisation and the elaboration of stressful or traumatic experiences could be highly beneficial for treating EDs and addictive behaviours, in young women particularly.

## Data availability statement

The datasets generated during and/or analysed during the current study are not publicly available due to confidentially. Requests to access the datasets should be directed to lauramacia@deusto.es.

## Ethics statement

The studies involving human participants were reviewed and approved by The Institutional Review Board of the University of Deusto (ETK-17/20–21). The patients/participants provided their written informed consent to participate in this study.

## Author contributions

LM: drafting of the manuscript, introduction, discussion, and conclusion. JM: design and target setting of the manuscript and bibliography. PM: drafting supervision and drafting of the method. MH: data analysis and results. PJ: principal investigator and coordinator of the funded project II recruitment of the sample. AE: supervision, manuscript design, and team coordination (principal investigator of the team). All authors contributed to the article and approved the submitted version.

## Funding

We thank the Spanish Ministry of Health for institutional support. The research was funded by the Delegación del Gobierno para el Plan Nacional sobre Drogas (PNSD; Government Delegation for the National Plan on Drugs; Ref: 2020I007). The funders played no role in the study design, data collection and analysis, decision to publish, or preparation of the manuscript. The research is also supported by a predoctoral grant for training university teachers from the Spanish Ministry of Universities (FPU20/03045) and a post-doctoral grant from the Basque Government.

## Conflict of interest

The authors declare that the research was conducted in the absence of any commercial or financial relationships that could be construed as a potential conflict of interest.

The reviewer GT declared a past co-authorship with one of the authors, AE, to the handling Editor.

## Publisher’s note

All claims expressed in this article are solely those of the authors and do not necessarily represent those of their affiliated organizations, or those of the publisher, the editors and the reviewers. Any product that may be evaluated in this article, or claim that may be made by its manufacturer, is not guaranteed or endorsed by the publisher.
